# Proteomic approach used in the diagnosis of Riedel's thyroiditis: a case report

**DOI:** 10.1186/1752-1947-6-103

**Published:** 2012-04-05

**Authors:** Pietro Iacconi, Laura Giusti, Ylenia Da Valle, Federica Ciregia, Gino Giannaccini, Liborio Torregrossa, Agnese Proietti, Gianluca Donatini, Salvatore Mazzeo, Fulvio Basolo, Antonio Lucacchini

**Affiliations:** 1 Department of Surgery, Via Paradisa 2, University of Pisa, 56124 Pisa, Italy; 2 Department of Psychiatry, Neurobiology, Pharmacology and Biotechnology, Via Bonanno 6, University of Pisa, 56126 Pisa, Italy; 3 Department of Oncology, Transplants and New Medical Techniques, Radiodiagnostic Unit, Via Roma 55, University of Pisa, 56126 Pisa, Italy

## Abstract

**Introduction:**

Riedel's thyroiditis, a rare thyroid disease, can be difficult to diagnose prior to surgical removal and can be confused with malignancy both clinically and cytologically.

**Case presentation:**

We report the case of a 72-year-old Caucasian woman who presented with a goiter, which showed a rapid increase in size at ultrasound check, suggesting malignancy. Because of inconclusive cytology, a total thyroidectomy was performed. Fine-needle aspiration of the removed thyroid was processed by two-dimensional electrophoresis, and the proteome was compared with both anaplastic cancer and control samples. Significant differentially expressed protein spots were identified by Western blot analysis by using specific antibodies.

**Conclusions:**

The protein pattern of Riedel's fine-needle aspiration revealed a superimposition with that of the control samples. The comparison of the protein pattern of Riedel's thyroiditis fine-needle aspiration with that of anaplastic cancer showed evidence of a different expression of ferritin heavy chains, ferritin light chains, and haptoglobins, as previously reported in thyroid cancers. Therefore, we performed Western blot analysis of these proteins and validated that their expression levels were low or absent in Riedel's thyroiditis and control samples despite the high concentrations present in fine-needle aspiration anaplastic samples. The concurrent absent or low expression levels of haptoglobin, ferritin light chain, and ferritin heavy chain in Riedel's thyroiditis fine-needle aspiration samples strongly indicate the benign nature of the thyroid lesion. These results suggest the potential applicability of fine-needle aspiration proteome analysis for Riedel's thyroiditis diagnosis.

## Introduction

Riedel thyroiditis is a rare chronic inflammatory disease of the thyroid gland and is characterized by the invasion of the thyroid gland and surrounding structures by dense fibrous tissues [1,2]. The operative incidence at the Mayo Clinic (Rochester, MN, USA) was 0.06%, and the overall incidence in out-patients was 1.6 per 100,000 [1]. Riedel's thyroiditis is most often seen in women. It is associated with other fibrous inflammatory processes such as retroperitoneal fibrosis, orbital pseudotumor, mediastinal fibrosis, sclerosing colangitis, and fibrosis in other organ systems [2]. Recently, Shahi and colleagues [3] reported the difficulty in distinguishing this disease from aggressive anaplastic thyroid cancer in a case of Riedel's thyroiditis.

In this report, we describe a rare case of Riedel's thyroiditis in which the diagnosis was obtained only after surgery. The study of proteome of fine-needle aspiration (FNA) fluid is a useful tool in distinguishing non-malignant from malignant thyroid lesions [4,5]. Thus, we demonstrate that the proteomic analysis of FNA of the patient with Riedel's thyroiditis strongly suggests its ability to assist in the differential diagnosis of Riedel's disease.

## Case presentation

**Figure 1:**
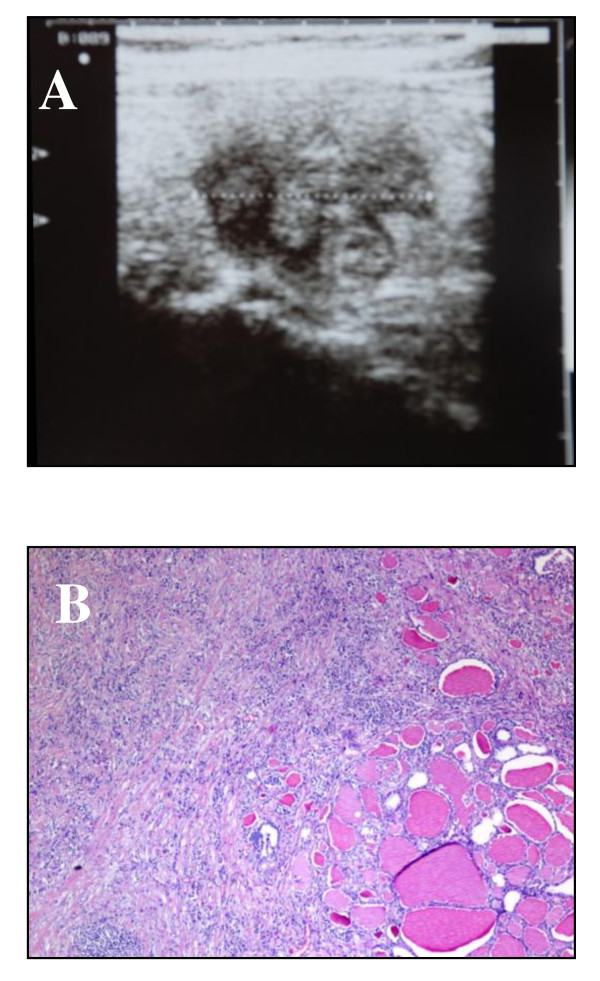
**Ultrasound and Histological images**. (A) Grey-scale ultrasound image of our patient's neck. The image was taken with commercially available 7.5 MHz linear probes and shows a multi-nodular goiter with a right-lobe hypoechoic nodule (about 2 cm in diameter) that has an irregular outline. (B) Invasive fibrous thyroiditis (Riedel's disease). The thyroid gland is replaced by dense keloid-like bands of fibrous tissue with mixed chronic inflammatory cells (lymphocytes and plasma cells).

A 72-year-old Caucasian woman from Southern Italy was admitted with a 15-year history of multi-nodular goiter to our hospital. She did not undergo therapy. The thyroid function test results were in the normal range. There was no family history of thyroid or autoimmune diseases. An ultrasound scan of the neck confirmed a multi-nodular goiter with a right-lobe hypoechoic nodule, which was stone-hard on palpation (Figure 1A). The nodule showed a hypovascular pattern at color Doppler sonography and a high resistance at the penetration of a 22-gauge needle. Cytological analysis of FNA samples was not useful for the diagnosis.

Because anaplastic carcinoma was suspected, our patient was submitted to a total thyroidectomy followed by a histological examination (Figure 1B). Surgical resection of the thyroid gland was difficult since the right lobe was markedly fibrotic and had dense capsular adhesions. However, it was possible to visualize the recurrent nerve, and a total thyroidectomy was performed even though there was an extension of a fibro-inflammatory infiltrate into the adjacent skeletal muscle and the adipose tissue. After thyroidoctomy, an FNA with a 23-gauge needle was performed on the nodule (5.2 × 4.2 × 2.2 cm) of the right lobe and on a normal portion of the thyroid tissue of the left lobe, as previously described [5]. FNA samples were processed to obtain protein pellets [5], proteomic analysis was performed by combining two-dimensional electrophoresis (2-DE), and mass spectrometry and Western blot (WB) analysis was carried out to validate protein expression. The procedures of 2-DE analysis [5,6], MALDI-TOF-TOF (matrix-assisted laser desorption/ionization time-of-flight time-of-flight) analysis [7], protein identification [8], and WB analysis [9] are reported in the Methods section of Additional file 1.

In addition to processing the Riedel's thyroiditis and the normal counterpart samples, we processed FNA samples (one man and two women who had a mean age of 65.3 years ± a standard deviation of three and a half years) of anaplastic lesions and the respective normal counterparts. An informed consensus was obtained for diagnostic or clinical purposes, and the study was approved by the local ethics committee.

**Figure 2:**
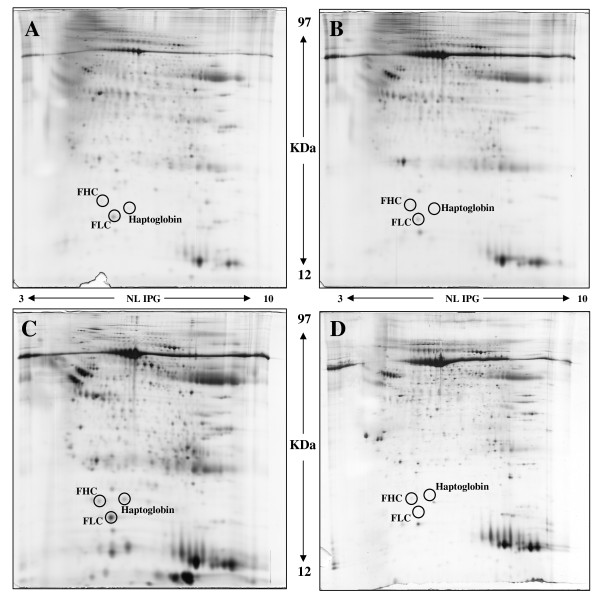
**Fine-needle aspiration (FNA) proteome analysis**. Two-dimensional electrophoresis (2-DE) images of Riedel's thyroiditis (A) and the normal counterpart (B) are shown. Representative FNA profiles of both anaplastic (C) and normal (D) samples are shown.

**Figure 3:**
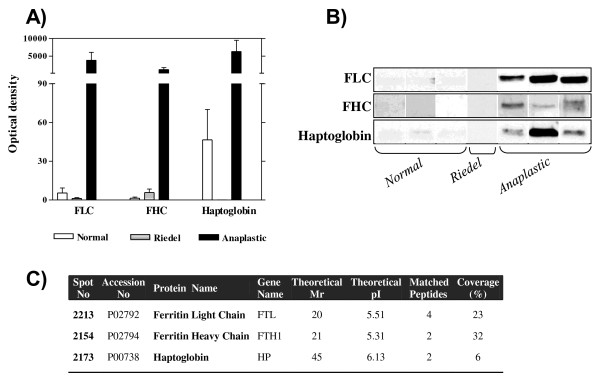
**Immunoblot analysis of ferritin light chain (FLC), ferritin heavy chain (FHC), and haptoglobin**. Western blot is representative of three fine-needle aspiration (FNA) profiles of anaplastic (n = 3) and normal (n = 3) samples analyzed in triplicate and of the Riedel's thyroiditis (RT) FNA performed in triplicate (B). Densitometry of the blots is shown in (A). Statistically significant differences were observed between anaplastic and both normal (P < 0.05) and RT (P < 0.05) samples. No statistically significant differences were observed between normal and RT samples. (C) The table shows the mass spectrometry parameters for the proteins identified.

Figure 2 shows the 2-DE images of Riedel's thyroiditis (A) and the normal counterpart (B). The representative 2-DE images of both anaplastic (C) and normal (D) samples are shown for comparison. From quantitative and qualitative points of view, the Riedel's thyroiditis images are similar to those of normal samples, although significant differences were observed with respect to the images of anaplastic proteome. The main differences had to do with the expression of the three proteins that we had previously found to be upregulated in the thyroid cancer [5]. These proteins were haptoglobin, ferritin light chain, and ferritin heavy chain. Mass spectrometry confirmed the identification of these proteins in our FNA samples (Figure 3C), and WB analysis, in which specific antibodies were used, validated the significant change of expression between Riedel's thyroiditis and anaplastic lesions (Figure 3B). Very low expressions for haptoglobin, ferritin light chain, and ferritin heavy chain were found in both Riedel's thyroiditis and normal samples compared with those exhibiting anaplastic cancer, in which increases of more than 100, more than 500, and more than 300 were observed (Figure 3A). Statistically significant differences in the immunoreactive bands were calculated by Mann-Whitney test: after comparison of protein level expression between Riedel's thyroiditis and anaplastic FNA, the P values were 0.037, 0.035, and 0.047 for haptoglobin, ferritin light chain, and ferritin heavy chain, respectively. No significant differences between Riedel's thyroiditis and normal samples were observed. P values were 0.37, 0.73, and 0.11 for haptoglobin, ferritin light chain, and ferritin heavy chain, respectively.

## Discussion

Riedel's thyroiditis is a progressive disease with a good prognosis after surgery. This rare fibrosclerotic infiltrative thyroid disorder of unclear etiology expands outside the thyroid capsule and is sometimes part of multi-focal fibrosclerotic lesions (retroperitoneal, retro-orbital, and mediastinal) [1,10-12]. Surgical resection may be difficult, and there is an elevated risk of injury of neck structures such as the recurrent laryngeal nerve [13]. Some authors have treated it with tamoxifen [14], whereas the majority of authors have reported the beneficial effects of steroids [3,13].

The main differential diagnosis is invasive thyroid cancer. Owing to the familial occurrence of multiple fibrosclerotic lesions, which include Riedel's thyroiditis, some authors have suggested that it is a genetically transmitted disease [15]. The clinical presentation mimics that of invasive thyroid carcinoma [12]. Laboratory findings in Riedel's thyroiditis are non-specific, but in some patients the erythrocyte sedimentation rate is elevated. Autoantibodies may or may not be positive [10]. Owing to the dense fibrosis that precludes adequate aspiration, FNA is rarely diagnostic [16].

Recent advances in proteomic technologies have allowed simultaneous identification of several proteins, leading to biomarker discoveries and a better understanding of the disease processes. In previous studies [4,5], we found that haptogloblin, ferritin light chain, and ferritin heavy chain were differentially expressed in the FNA of thyroid cancers. Therefore, in this study, we explored whether the level of expression of these proteins in FNA of Riedel's thyroiditis was comparable to the level of expression of the FNA of normal thyroid tissues or anaplastic cancer lesions. Interestingly, in addition to detecting the strong similarity between Riedel's thyroiditis and normal FNA pattern profiles, we detected very low levels of haptogloblin, ferritin light chain, and ferritin heavy chain protein expressions in Riedel's thyroiditis.

## Conclusions

This case demonstrated, for the first time, that the FNA proteome can help us in the differential diagnosis of Riedel's thyroiditis with respect to anaplastic thyroid lesions. We suggest, in particular, that the concurrent absent or low expression levels of haptoglobin, ferritin light chain, and ferritin heavy chain strongly indicate the benign nature of the lesion. Finally, this report suggests an active role of proteomic analysis in the diagnosis of Riedel's thyroiditis.

## Abbreviations

2-DE: two-dimensional electrophoresis; FNA: fine-needle aspiration; WB: Western blot.

## Consent

Written informed consent was obtained from the patient for publication of this case report and any accompanying images. A copy of the written consent is available for review by the Editor-in-Chief of this journal.

## Competing interests

The authors declare that they have no competing interests.

## Authors' contributions

PI and AL helped to design the study, coordinate the research, analyze data, and write the manuscript. LG helped to design the study, coordinate the research, analyze data, write the manuscript, and carry out proteomic analysis. FB and GG participated in the design and coordination of study and helped to draft the manuscript. LT and AP carried out cytological and immunohystochemical analysis. YDV and FC helped to carry out proteomic analysis. SM carried out instrumental diagnostic analysis. GD carried out statistical analysis. All authors read and approved the final manuscript.

## Supplementary Material

Additional file 1 **Methods and analysis**. Click here for file
