# Supplementary material for: Proteomic approach used in the diagnosis of Riedel's thyroiditis: a case report
Source: J Med Case Rep. 2012 Apr 5;6:103. doi: 10.1186/1752-1947-6-103 (PMC3349472; doi:10.1186/1752-1947-6-103)
Supplement: Additional file 1 — Methods and analysis. [file 1752-1947-6-103-S1.DOC]

*2DE analysis*

Isoelectrofocusing (IEF) was carried out by using 18 cm Immobiline Dry-Strips (GE Healthcare) with a non linear (NL), pH 3-10, gradient. Samples were treated as previously described [5] Briefly, 150 µg of protein of each samples was filled up to 350 µl with rehydration buffer supplemented with 1 % (v/v) pharmalytes, pH 3-10 IEF was performed at 16°C on a Ettan IPGphor II apparatus (GE Health Care) and proteins were then focused for up to 70000 Vh at a maximum of 8000 V. After IEF the strips were equilibrated and applied to the top of 12.5% SDS-PAGE gels , running at 40 mA/gel and 10°C for about 6 h using the PROTEAN-II Multi Cell system (Bio-Rad).

*Proteomic analysis*

Protein visualisations were obtained by silver staining [6] and a comparison of images was performed by using the 2D Same Spots software (Progenesis). The gels were performed in triplicate. The significance of the differences (p-value <0.05) was calculated using the Mann-Whitney test. Spots of interest were cut and processed by performing a MALDI-TOF-TOF analysis

*MALDI-TOF-TOF analysis and protein identification*

A fresh destain solution was prepared mixing 30 mM potassium ferricyanide solution and 100 mM sodium thiosulfate solution in a 1:1 ratio. Add 100 μl of the destain solution to each eppendorf and incubate with occasional vortexing until the gel is completely destained (about 5-10 min). Then the gel pieces were washed two times with 100 μl of distilled water for 10 min. Subsequently, gel pieces were rehydrated with 100 μl of 50 mM ammonium bicarbonate for 10 min and dehydrated with 100 μl of 50 mM ammonium bicarbonate in 30% for 10 min.

Gel pieces were then dried for 30 minutes in a Hetovac vacuum centrifuge (HETO, Allerod, Denmark). Dried pieces of gel were subjected to protein digestion by trypsin and peptide extraction.

MS and MS/MS analysis of peptides were performed with a 4800 Proteomics Analyzer MALDI-TOF/TOF mass spectrometer (Applied Biosystems, Framingham, MA, USA) according to the tuning procedures suggested by the manufacturer. Peak lists were generated with the Launch peak to MASCOT tools. with the following settings: for the MS data, mass range 850– 4000, peak density of maximum 20 peaks per 100 Da, minimal S/N ratio of 15, minimal area of 250, max peak 50; for the MS/MS data, mass range 60–2000; peak density of maximum 50 peaks per 200 Da, minimal S/N ratio of 5, minimal area of 20, and maximum number of peak set at 200. Such acquired MS and MS/MS data were compared to the database using MASCOT search engine (http://www.matrixscience). In MASCOT, the combined PMF and MS/MS search was performed on uniprot_sptr_15.10-03-Nov-2009 database (selected for Homo sapiens, 98529 entries). Search settings allowed one missed cleavage with the trypsin enzyme selected, one fixed modification (carboxymethylated cysteine) and a variable modification (oxidation of methionine).

Scaffold (version Scaffold_3_00_03, Proteome Software Inc., Portland, OR) was used to validate MS/MS based peptide and protein identifications. Peptide identifications were accepted if they could be established at greater than 95.0 % probability as specified by the Peptide Prophet algorithm [7] Protein identifications were accepted if they could be established at greater than 95.0 % probability and contained at least 2 identified peptides. Protein probabilities were assigned by the Protein Prophet algorithm [8]. Proteins that contained similar peptides and could not be differentiated based on MS/MS analysis alone were grouped to satisfy the principles of parsimony.

*Western Blot Analysis*

Western Blot analysis of ferritin heavy chain (FHC), ferritin light chain (FLC), and haptoglobin was performed essentially as previously described [9]. Briefly, aliquot of proteins (20 μg), from FNA samples (RT, anaplastic and normal), were loaded on 12% acrylamide gels and processed [9]. For the protein detection the following antibodies were used: rabbit polyclonal anti-FHC (dilution 1:200) (Santa Cruz Biotecnology CA, USA), rabbit polyclonal anti-FLC (dilution 1:2000) (Abcam, UK) and rabbit polyclonal anti-haptoglobin (dilution 1:5000) (Abcam, UK). All the experiments were carried out in triplicate. For each tested protein, the optical density of specific immunoreactive bands was determined (Image J software), and the resulting mean values ± SD were compared (Mann-Withney test).
